# Ascertainment of vaccination status by self‐report versus source documentation: Impact on measuring COVID‐19 vaccine effectiveness

**DOI:** 10.1111/irv.13023

**Published:** 2022-07-11

**Authors:** Meagan Stephenson, Samantha M. Olson, Wesley H. Self, Adit A. Ginde, Nicholas M. Mohr, Manjusha Gaglani, Nathan I. Shapiro, Kevin W. Gibbs, David N. Hager, Matthew E. Prekker, Michelle N. Gong, Jay S. Steingrub, Ithan D. Peltan, Emily T. Martin, Raju Reddy, Laurence W. Busse, Abhijit Duggal, Jennifer G. Wilson, Nida Qadir, Christopher Mallow, Jennie H. Kwon, Matthew C. Exline, James D. Chappell, Adam S. Lauring, Adrienne Baughman, Christopher J. Lindsell, Kimberly W. Hart, Nathaniel M. Lewis, Manish M. Patel, Mark W. Tenforde, IVY Network Investigators

**Affiliations:** ^1^ CDC COVID‐19 Response Team Atlanta Georgia USA; ^2^ Vanderbilt University Medical Center Nashville Tennessee USA; ^3^ University of Colorado School of Medicine Aurora Colorado USA; ^4^ University of Iowa Iowa City Iowa USA; ^5^ Baylor Scott & White Health Temple Texas USA; ^6^ Texas A&M University College of Medicine Temple Texas USA; ^7^ Beth Israel Deaconess Medical Center Boston Massachusetts USA; ^8^ Wake Forest University Baptist Medical Center Winston‐Salem North Carolina USA; ^9^ Johns Hopkins Hospital Baltimore Maryland USA; ^10^ Hennepin County Medical Center Minneapolis Minnesota USA; ^11^ Montefiore Healthcare Center Albert Einstein College of Medicine Bronx New York USA; ^12^ Baystate Medical Center Springfield Massachusetts USA; ^13^ Intermountain Medical Center and University of Utah Salt Lake City Utah USA; ^14^ University of Michigan School of Public Health Ann Arbor Michigan USA; ^15^ Oregon Health & Science University Hospital Portland Oregon USA; ^16^ Emory University School of Medicine Atlanta Georgia USA; ^17^ Cleveland Clinic Cleveland Ohio USA; ^18^ Stanford University School of Medicine Palo Alto California USA; ^19^ Ronald Reagan‐UCLA Medical Center Los Angeles California USA; ^20^ University of Miami Miami Florida USA; ^21^ Washington University St. Louis Missouri USA; ^22^ Ohio State University Wexner Medical Center Columbus Ohio USA; ^23^ University of Michigan School of Medicine Ann Arbor Michigan USA

**Keywords:** concordance, COVID‐19, registry, self‐report, vaccine effectiveness

## Abstract

**Background:**

During the COVID‐19 pandemic, self‐reported COVID‐19 vaccination might facilitate rapid evaluations of vaccine effectiveness (VE) when source documentation (e.g., immunization information systems [IIS]) is not readily available. We evaluated the concordance of COVID‐19 vaccination status ascertained by self‐report versus source documentation and its impact on VE estimates.

**Methods:**

Hospitalized adults (≥18 years) admitted to 18 U.S. medical centers March–June 2021 were enrolled, including COVID‐19 cases and SARS‐CoV‐2 negative controls. Patients were interviewed about COVID‐19 vaccination. Abstractors simultaneously searched IIS, medical records, and other sources for vaccination information. To compare vaccination status by self‐report and documentation, we estimated percent agreement and unweighted kappa with 95% confidence intervals (CIs). We then calculated VE in preventing COVID‐19 hospitalization of full vaccination (2 doses of mRNA product ≥14 days prior to illness onset) independently using data from self‐report or source documentation.

**Results:**

Of 2520 patients, 594 (24%) did not have self‐reported vaccination information to assign vaccination group; these patients tended to be more severely ill. Among 1924 patients with both self‐report and source documentation information, 95.0% (95% CI: 93.9–95.9%) agreement was observed, with a kappa of 0.9127 (95% CI: 0.9109–0.9145). VE was 86% (95% CI: 81–90%) by self‐report data only and 85% (95% CI: 81‐89%) by source documentation data only.

**Conclusions:**

Approximately one‐quarter of hospitalized patients could not provide self‐report COVID‐19 vaccination status. Among patients with self‐report information, there was high concordance with source documented status. Self‐report may be a reasonable source of COVID‐19 vaccination information for timely VE assessment for public health action.

## 1 INTRODUCTION

In December 2020, two COVID‐19 mRNA vaccines (Pfizer‐BioNTech and Moderna) received Emergency Use Authorization (EUA), and by early 2022, both COVID‐19 mRNA vaccines received full Food and Drug Administration (FDA) approval for individuals ≥18 years of age.^1^, ^2^, ^3^ As of April 2022, an estimated 255 million persons in the United States had received one or more doses of a COVID‐19 vaccine.^1^ Timely post‐marketing evaluations of COVID‐19 vaccine effectiveness (VE) have been critical to understand the real‐world protection provided by COVID‐19 vaccination and to inform public health measures throughout the pandemic.^4^ Observational studies employing case‐control designs typically infer protection by comparing the odds of antecedent COVID‐19 vaccination in cases versus controls. To reduce the potential underestimation of VE introduced by vaccination reporting bias, COVID‐19 vaccination status needs to be accurately classified.

To ascertain COVID‐19 vaccination status, post‐marketing evaluations have used various data sources typically categorized as self‐report or source documentation of vaccination. In the United States, documented sources of vaccination typically include computerized immunization information systems (IIS) (also known as vaccine registries), electronic medical records, pharmacy records, occupational health records, and vaccination record cards.^5^, ^6^, ^7^, ^8^ For COVID‐19 vaccination providers, reporting of vaccination to IIS is generally required.^9^, ^10^ However, the compilation and subsequent analysis of VE data can be compromised by receipt of vaccines in a different jurisdiction than SARS‐CoV‐2 testing or hospitalization, loss of vaccination cards, timeliness of reporting to electronic systems, and lack of access to IIS and other documented sources among study investigators.

Therefore, self‐report of COVID‐19 vaccination, defined as the patient or a proxy verbally providing a history of prior vaccination, might serve as a useful source of COVID‐19 vaccination data during the pandemic and facilitate timely evaluations of VE. However, relying on self‐report of COVID‐19 vaccination could also result in misclassification of vaccination status through recall bias, social desirability bias,^11^ or other mechanisms. To inform the use of self‐report versus source documentation for estimating VE against COVID‐19‐related hospitalization, we evaluated concordance of COVID‐19 vaccination status ascertained from these sources among hospitalized adults in a multistate VE surveillance network during the first few months of the COVID‐19 vaccine roll‐out.

## 2 METHODS

### 2.1 Participants and setting

To evaluate ascertainment of COVID‐19 vaccination status by self‐report versus source documentation, we used data from the Influenza and Other Viruses in the Acutely Ill (IVY) network, a multistate network that conducts analyses of real‐world VE against COVID‐19 hospitalizations among adults.^12^ In brief, this analysis included hospitalized adults (aged ≥18 years) from 18 academic medical centers in 16 states with admission dates from March 11 to June 6, 2021. Trained medical abstractors reviewed hospital admission logs or medical records daily at participating sites to identify hospitalized patients who had received clinical testing for acute SARS‐CoV‐2 infection. Patients who tested positive for SARS‐CoV‐2 by reverse transcription (RT)‐PCR and met criteria for COVID‐19‐like illness were included as COVID‐19 cases. We used two control groups, which were combined in this analysis. Test‐negative controls included adults hospitalized with COVID‐19‐like illness who tested negative for SARS‐CoV‐2 by RT‐PCR. We also included a secondary control group without COVID‐19‐like illness who tested negative for SARS‐CoV‐2 by RT‐PCR (syndrome‐negative controls).

### 2.2 COVID‐19 vaccination history

Data were collected specifically on COVID‐19 vaccination, and no other vaccinations were assessed. Details about COVID‐19 vaccination, including dates, locations, vaccine product (i.e., Pfizer‐BioNTech, Moderna, or Johnson & Johnson's [J&J] Janssen COVID‐19 vaccines or other including vaccines not authorized for use in the United States), and lot number, were collected by combining data from interviews and source documentation. Self‐reported data were collected through structured interviews with patients or their proxies (if patients were unable to be interviewed). If COVID‐19 vaccination record cards were available from patients or proxies, information on the card was collected, and additional vaccine verification was not performed. For patients without COVID‐19 vaccine record cards available, queries of source documentation were performed whether patients or proxy reported receiving or not receiving COVID‐19 vaccination. Source documentation included hospital electronic medical records (EMRs), local IIS reviewed at the time of interview and again approximately 28 days later, and vaccine records requested from clinics and pharmacies. If a vaccination card was available during the interview (N = 145/2520), the vaccine card information was considered both self‐reported and documented as it was provided by the patient or proxy.

### 2.3 COVID‐19 vaccination status comparison by data source

We compared COVID‐19 vaccination status determined through self‐report with vaccination status determined through source documentation. To mirror the vaccination groups used in previous VE analyses using these data,^12^ we classified vaccination status for COVID‐19 mRNA vaccines into 4 groups:

1. Unvaccinated: No vaccine doses received by illness onset;
2. Vaccinated but unprotected: One dose of vaccine received 0–13 days prior to illness onset, including for the one‐dose Janssen vaccine (i.e., likely non‐optimal immunity);
3. Partially vaccinated: One dose of a two‐dose vaccine series received ≥14 days prior to illness onset or two doses in a two‐dose series received with the second dose <14 days prior to illness onset, and
4. Fully vaccinated: Second dose of a two‐dose mRNA vaccine series or first and only dose of the one‐dose Johnson & Johnson (Janssen) vaccine, received ≥14 days prior to illness onset.

To compare COVID‐19 vaccination status by self‐report and source documentation, we estimated the percent agreement and unweighted kappa with 95% confidence intervals (CIs). To be considered vaccinated by self‐report, the patient or proxy needed to provide both a date and location of vaccination. For patients or proxies who reported vaccination but did not know the vaccine product, we assumed the patient received an mRNA vaccine as these vaccine products accounted for most vaccines administered in the United States during the surveillance period.^1^ We performed unadjusted logistic regression to identify any associations between COVID‐19 vaccination status discordance (self‐report versus source documentation) and any of the following: age, sex, race/ethnicity, U.S. Census region, health insurance, interview of patient versus other (proxy, mix of patient or proxy, or unspecified), education (some college or more versus less than college), or employment status. Syndrome‐negative controls did not have illness onset dates because they were admitted to the hospital for reasons other than an acute respiratory illness; for these patients, we used the date of hospital admission as the reference date of illness onset for determining vaccination group.

Patients who had unknown COVID‐19 vaccination status or could not provide self‐reported dates of vaccination or could not be assigned to a vaccination group were excluded from concordance analyses but were compared by demographic characteristics with patients whose vaccination status and date could be classified by either self‐report or source documentation. Additionally, patients who participated in blinded COVID‐19 vaccine randomized clinical trials were excluded.

### 2.4 COVID‐19 VE comparison

In prior analyses of mRNA COVID‐19 VE,^5^, ^12^, ^13^, ^14^ we primarily considered a patient vaccinated based on source documentation but accepted self‐report of vaccination when vaccination status from documented sources was missing. In the case of vaccination dates or products that differed between self‐report and documented sources, data from documented sources were used. For this analysis, we compared VE estimates obtained using (1) self‐reported data only or (2) source documentation data only, with the goal of evaluating whether estimates were similar across data sources.

VE was calculated by comparing the odds of being fully vaccinated versus unvaccinated in case patients and controls (VE = (1 – adjusted odds ratio) × 100%). VE for partially vaccinated versus unvaccinated was similarly assessed. Models were adjusted for potential confounders including geographic region (Health and Human Services region of the admitting hospital), calendar time of admission in biweekly intervals, continuous age, sex, and race/ethnicity. For VE estimates, we excluded patients tested for SARS‐CoV‐2 >10 days after illness onset, hospitalized >14 days after illness onset, who received a non‐mRNA vaccine product (for which sample size was insufficient for analysis), for whom the vaccine product was not known, and who were enrolled in the syndrome‐negative control group but later tested positive for SARS‐CoV‐2 infection.

SAS 9.4 (Cary, NC) was used for statistical analyses. Significance thresholds of *p* < 0.05 were applied for all analyses. This program was determined to be a public health surveillance activity by each participating site and CDC and conducted in a manner consistent with applicable federal law and CDC policy (45 C.F.R. part 46.102(l)(2), 21 C.F.R. part 56; 42 U.S.C. §241(d); 5 U.S.C. §552a; 44 U.S.C. §3501 et seq.). The IVY Network has previously reported COVID‐19 VE estimates during this surveillance period^12^, ^15^; although VE estimates are also reported in this manuscript, the objective was primarily to compare vaccine status and similarity of estimated VE across data sources.

## 3 RESULTS

### 3.1 Patient characteristics of those included in vaccination concordance analysis

**FIGURE 1:**
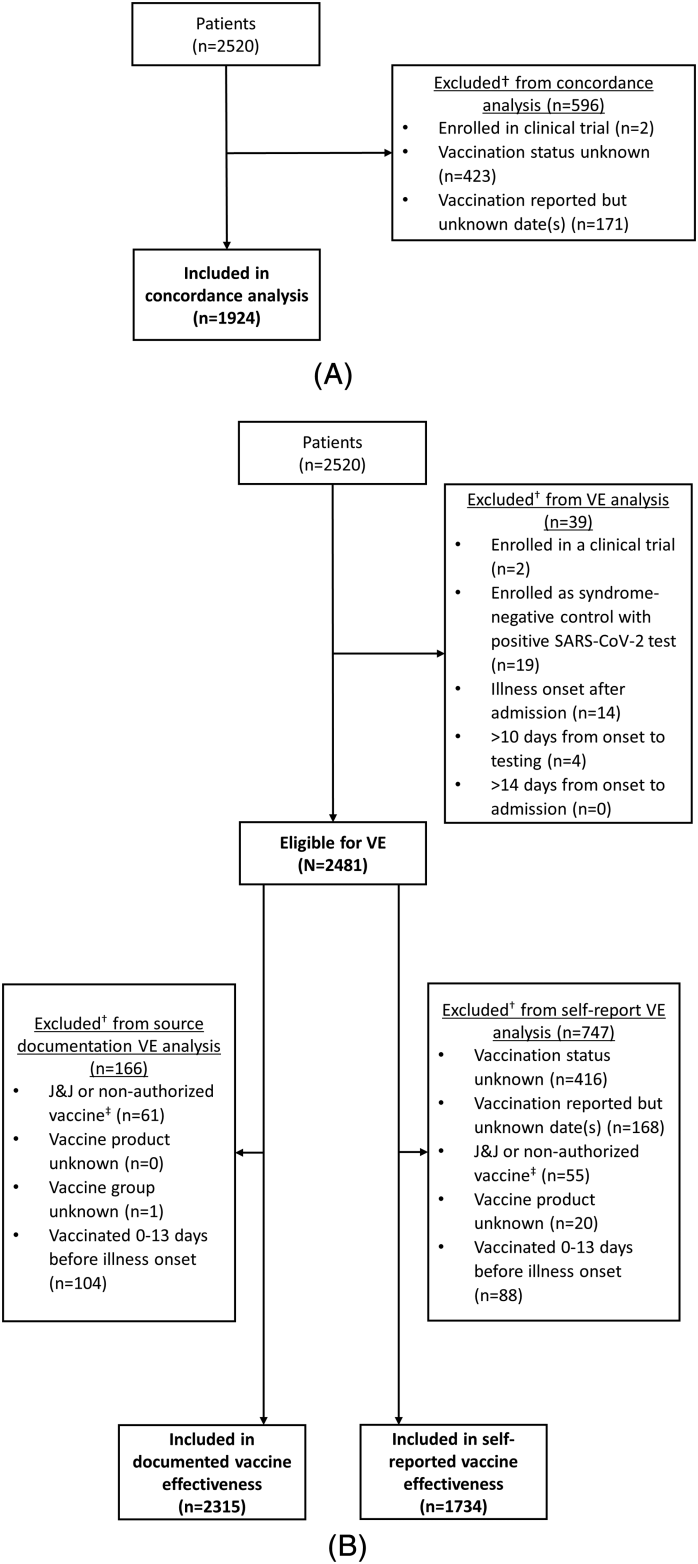
Flow diagram of hospitalized patients in concordance and vaccine effectiveness analysis, 18 U.S. medical centers, March 11–June 6, 2021. Panel 1A. Flow diagram of patients in concordance analysis. ^†^Exclusion categories are mutually exclusive. Panel 1B. Flow diagram of patients in vaccine effectiveness analysis. Abbreviations: VE, vaccine effectiveness; J&J, Janssen's Johnson & Johnson. ^†^Exclusion categories are mutually exclusive. ^‡^Includes patients with mixed vaccine products (e.g., one dose of Moderna and one dose of Pfizer‐BioNTech)

Of 2520 hospitalized cases and controls, 596 (24%) were excluded from the COVID‐19 vaccination status concordance analysis because self‐report data were missing or incomplete (n = 594) or the patient was previously enrolled in a blinded COVID‐19 vaccine trial (n = 2) (Figure 1A). Compared with patients who were able to provide a self‐reported vaccination history, patients with missing self‐reported vaccination data were more likely to be older (median age of 61 vs. 58 years), admitted to the intensive care unit (ICU) (25% vs. 17%), and of Hispanic/Latino race/ethnicity (17% vs. 14%) (all *p* < 0.01) (Table S1).

Of 1924 patients included in the concordance analysis, for whom both self‐reported and source documentation data were available, proportions allocated to different vaccination categories were similar based on self‐report and source documentation (Table 1). A total of 792 (41%) were vaccinated with at least one COVID‐19 vaccine dose by self‐report and 756 (39%) by documented vaccination. Among those with at least one vaccine dose, 112 (14%) received a first dose <14 days before symptom onset (i.e., were vaccinated but unprotected) by self‐report and 102 (13%) by source documentation; 193 (24%) were partially vaccinated by self‐report and 176 (23%) by source documentation; and 487 (61%) were fully vaccinated by self‐report and 478 (63%) by documented vaccination. Vaccine products reported were similar by either method. Using source documentation, all vaccinated patients had a manufacturer recorded in the registry, electronic medical record (EMR), or other vaccination source. A total of 18 (2%) patients were unable to self‐report the manufacturer of the vaccine, and one (<1%) patient self‐reported receipt of a COVID‐19 vaccine not authorized for use in the United States (Oxford/AstraZeneca or Covishield).

**TABLE 1:** Characteristics of vaccinated and unvaccinated hospitalized adults in concordance analysis of self‐reported versus documented COVID‐19 vaccination status, 18 U.S. medical centers, March 11–June 6, 2021

Characteristic	COVID‐19 Vaccination Status			
	Self‐Report		Source Documentation	
	Vaccinated (≥1 dose)	Unvaccinated	Vaccinated (≥1 dose)	Unvaccinated
Total (n = 1924)	n = 792	n = 1132	n = 756	n = 1168
Age in years, median (IQR)	63 (52–72)	55 (42–64)	64 (54–73)	55 (42–64)
Age group, no./total no. (%)				
18‐49 years	160/788 (20)	442/1130 (39)	141/752 (19)	461/1166 (40)
50‐64 years	259/788 (33)	418/1130 (37)	251/752 (33)	426/1166 (37)
≥65 years	369/788 (47)	270/1130 (24 16 )	360/752 (48)	279/1166 (24)
Female sex, no./total no. (%)	396/791 (50)	554/1131 (49)	386/755 (51)	564/1167 (48)
Race/ethnicity, no./total no. (%)				
White, non‐Hispanic	526/792 (66)	585/1132 (52)	506/756 (67)	605/1168 (52)
Black, non‐Hispanic	138/792 (17)	292/1132 (2617)	132/756 (17)	298/1168 (26)
Hispanic	87/792 (11)	184/1132 (16)	80/756 (11)	191/1168 (16)
Other, non‐Hispanic	31/792 (4)	61/1132 (5)	30/756 (4)	62/1168 (5)
Unknown	10/792 (1)	10/1132 (1)	8/756 (1)	12/1168 (1)
Census region, no./total no. (%)				
East	122/792 (15)	174/1132 (15)	119/756 (16)	177/1168 (15)
South	257/792 (32)	487/1132 (43)	244/756 (32)	500/1168 (43)
Midwest	258/792 (33)	226/1132 (20)	249/756 (33)	235/1168 (20)
West	155/792 (20)	245/1132 (22)	144/756 (19)	256/1168 (22)
Health insurance, no./total no. (%)	766/790 (97)	1031/1126 (92)	732/753 (97)	1065/1163 (92)
Some college or more, no./total no. (%)	448/714 (63)	450/971 (46)	428/684 (63)	470/1001 (47)
Employed, no./total no. (%)	236/755 (31)	401/1050 (38)	217/722 (30)	420/1083 (39)
Interviewee type, no./total no. (%)				
Patient	677/792 (85)	977/1132 (86)	‐‐‐	‐‐‐
Proxy	44/792 (6)	77/1132 (7)	‐‐‐	‐‐‐
Mix patient/proxy	20/792 (3)	18/1132 (2)	‐‐‐	‐‐‐
Not specified	51/792 (6)	60/1132 (5)	‐‐‐	‐‐‐
Vaccine status^a^, no./total no. (%)				
Unvaccinated	‐‐‐	1132/1132 (100)	‐‐‐	1168/1168 (100)
First dose 0–13 days before onset	112/792 (14)	‐‐‐	102/756 (13)	‐‐‐
Partially vaccinated	193/792 (24)	‐‐‐	176/756 (23)	‐‐‐
Fully vaccinated	487/792 (61)	‐‐‐	478/756 (63)	‐‐‐
Vaccine type, if vaccinated, no./total no. (%)				
Pfizer‐BioNTech	409/792 (52)	‐‐‐	402/756 (53)	‐‐‐
Moderna	311/792 (39)	‐‐‐	307/756 (41)	‐‐‐
Mixed Pfizer‐BioNTech/Moderna	2/792 (<1)	‐‐‐	1/756 (<1)	‐‐‐
Janssen's Johnson & Johnson	51/792 (6)	‐‐‐	45/756 (6)	‐‐‐
Other (Oxford/AstraZeneca, Covishield)	1/792 (<1)	‐‐‐	0/756 (0)	‐‐‐
Unknown	18/792 (2)	‐‐‐	1/756 (<1)	‐‐‐
COVID‐19 RT‐PCR test result, no./total no. (%)				
Positive	178/792 (22)	621/1132 (55)	167/756 (22)	632/1168 (54)
Negative	614/792 (78)	511/1132 (45)	589/756 (78)	536/1168 (46)

Abbreviation: IQR, interquartile range.

^a^ COVID‐19 vaccination status included unvaccinated—defined as no receipt of any SARS‐CoV‐2 vaccine; partially vaccinated—defined as receipt of both doses of a two‐dose mRNA vaccine with the second dose received <14 days before illness onset; and fully vaccinated—defined as receipt of both doses of a two‐dose mRNA vaccine with the second dose received ≥14 days before illness onset.

### 3.2 Comparison of vaccination status by data source

**FIGURE 2:**
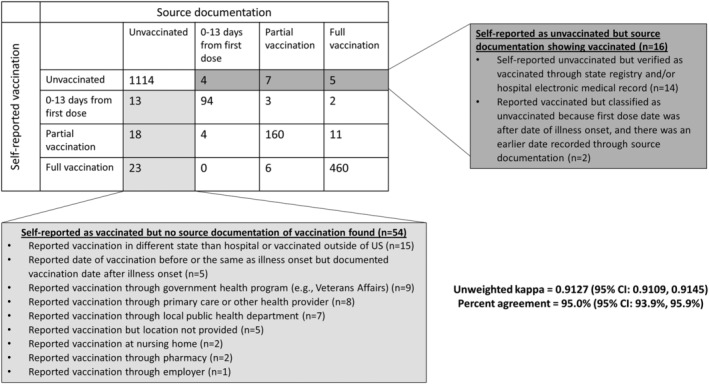
Comparison of self‐reported versus documented COVID‐19 vaccination status (n = 1924), 18 U.S. medical centers, March 11–June 6, 2021.^†^ Abbreviation: CI, confidence interval. ^†^COVID‐19 vaccination status included unvaccinated—defined as no receipt of any SARS CoV‐2 vaccine; partially vaccinated—defined as receipt of both doses of a two‐dose mRNA vaccine with the second dose received <14 days before illness onset; and fully vaccinated—defined as receipt of both doses of a two‐dose mRNA vaccine with the second dose received ≥14 days before illness onset

High agreement in COVID‐19 vaccination status by vaccination group was observed between the self‐report group and source documentation group. Overall, there was 95.0% (95% CI: 93.9–95.9%) agreement between sources within the four vaccination status categories, with an unweighted Kappa of 0.9127 (95% CI: 0.9109–0.9145) suggesting strong agreement between data sources (Figure 2). Fifty‐four (3%) patients had verbally reported receipt of one or more doses of a COVID‐19 vaccine without record of vaccination by source documentation, of which 15 (28%) reported COVID‐19 vaccination in a state or country different from where they were hospitalized, 9 (17%) reported vaccination through a military healthcare program, and 30 (56%) reported less common locations (Figure 2). In univariable regression models, female sex, having a high‐school education or less, and being unemployed were associated with discordance between self‐report and documented vaccination status (*p* < 0.05 for all). Age, race/ethnicity, census region of hospital, health insurance status, interview respondent (patient or proxy), and cohort designation were not associated with discordant vaccination status between self‐report and source documentation (*p* > 0.05 for all) (Table 2).

**TABLE 2:** Unadjusted odds ratios (ORs) of discordance between self‐reported and documented COVID‐19 vaccination status by demographic groups (n = 1924), 18 U.S. medical centers, March 11–June 6, 2021

Characteristic	Unadjusted OR (95% CI)	*p* value^a^
Age group		0.54
18–49 years	Reference	
50–64 years	0.97 (0.60–1.58)	
≥65 years	1.27 (0.75–2.15)	
Sex		0.01
Male	Reference	
Female	1.84 (1.20–2.82)	
Race/ethnicity		0.46
White, non‐Hispanic	Reference	
Black, non‐Hispanic	1.58 (0.89–2.81)	
Hispanic	0.97 (0.54–1.74)	
Other, non‐Hispanic	1.26 (0.45–3.54)	
Unknown	0.51 (0.12–2.26)	
Census region		0.46
East	Reference	
South	1.15 (0.62–2.15)	
Midwest	1.12 (0.57–2.20)	
West	0.77 (0.40–1.48)	
Health insurance		0.97
No	Reference	
Yes	1.02 (0.44–2.38)	
Interviewee type		0.89
Other (proxy, mix patient/proxy, not specified)	Reference	
Patient only	0.96 (0.53–1.74)	
Some college or more		0.02
No	Reference	
Yes	0.57 (0.36–0.90)	
Employed		0.02
No	Reference	
Yes	0.60 (0.39–0.92)	
Cohort		0.09
SARS‐CoV‐2 case	Reference	
Test‐negative control	0.90 (0.54–1.52)	
Syndrome‐negative control	0.60 (0.37–0.97)	

Abbreviation: CI, confidence interval.

^a^ Chi‐square test used for statistical comparison.

### 3.3 VE comparison

For VE analyses of mRNA vaccines, 2315 patients were eligible for inclusion in the VE analysis using documented COVID‐19 vaccine status only and 1734 using self‐reported vaccination status data only (Figure 1B). VE against COVID‐19‐associated hospitalization for full mRNA vaccination was 85% (95% CI: 81–89%) using vaccination status from source documentation only and 86% (95% CI: 81–90%) using vaccination status from self‐report only, with overlapping CIs (Table 3).

**TABLE 3:** Unadjusted and adjusted COVID‐19 vaccine effectiveness for self‐reported and source documentation, 18 U.S. medical centers, March 11–June 6, 2021

Vaccination status^a^	Vaccinated cases/total cases (%)	Vaccinated controls/total controls (%)	Unadjusted VE (95% CI)	Adjusted VE^b^ (95% CI)
Self‐report				
Full vaccination	59/672 (9)	383/884 (43)	87% (83–91%)	86% (81–90%)
Partial vaccination	44/657 (7)	134/635 (21)	73% (62–81%)	74% (62–82%)
Source documentation				
Full vaccination	80/897 (9)	521/1188 (44)	88% (84–90%)	85% (81–89%)
Partial vaccination	59/876 (7)	171/838 (20)	72% (62–79%)	74% (63–81%)

Abbreviations: CI, confidence interval; VE, vaccine effectiveness.

^a^ COVID‐19 vaccination status included unvaccinated—defined as no receipt of any SARS‐CoV‐2 vaccine; partially vaccinated—defined as receipt of both doses of a two‐dose mRNA vaccine with the second dose received <14 days before illness onset; and fully vaccinated—defined as receipt of both doses of a two‐dose mRNA vaccine with the second dose received ≥14 days before illness onset.

^b^ VE was estimated using logistic regression comparing the odds of being fully vaccinated versus being unvaccinated in case patients and control patients using the equation VE = 100 × (1 – adjusted odds ratio). Models were adjusted for date of hospital admission (biweekly intervals), U.S. Department of Health and Human Services region of hospital, age group (18–49, 50–64, ≥65 years), sex, and race/ethnicity (non‐Hispanic/Latino White, non‐Hispanic/Latino Black, Hispanic/Latino of any race, non‐Hispanic/Latino Other, or unknown).

## 4 DISCUSSION

There was high concordance between self‐reported and documented COVID‐19 vaccination in adults hospitalized shortly after COVID‐19 vaccine authorization from March–June 2021 in a multi‐state network. The similar VE and high concordance between the two sources provided further support for combining self‐reported and documented COVID‐19 vaccination to determine the final analytic vaccination status, an approach used for VE analyses conducted by several groups, including the IVY Network, US Flu VE Network, Hospitalized Adult Influenza Vaccine Effectiveness Network, Overcoming COVID‐19 Network, and the New Vaccine Surveillance Network.^12^, ^18^, ^19^, ^21^, ^22^, ^23^, ^24^ During the COVID‐19 pandemic, when source documentation may be unavailable in a timely manner, self‐report could be reliably used in hospitalized adult populations to generate COVID‐19 VE estimates for public health action.

This analysis demonstrated utility of both self‐report and documented COVID‐19 vaccination and the benefit of collecting data from both sources. For most other vaccinations, obtaining documented vaccination status can be time‐consuming, can lead to delays in estimating VE, and can vary in completeness based on hospital site and jurisdiction.^18^, ^20^ However, considerable efforts have been made to improve accuracy and timeliness of source documentation of COVID‐19 vaccination, including IIS, EMR records, and the extensive use of COVID‐19 vaccination cards.^25^ For the COVID‐19 vaccines, jurisdictional registries have near real‐time reporting and improved infrastructure increasing awareness and use of IIS among adult healthcare providers in emergency settings.^20^, ^25^ Further, because our study was among hospitalized adults, there are limitations in access to these patients. Seventeen percent of participants in our study were admitted to the ICU where access is limited, especially during the COVID‐19 pandemic.^11^ Further, 6% of respondents were proxies and may not know the vaccination history of the patient, and 5% of patients included in the concordance analysis were not able to be interviewed. Hospital‐based studies that rely solely on self‐report for vaccination status are at risk for missing vaccination data on a large proportion of patients, as illustrated by 24% of the population in this study not having self‐report data. Importantly, this missingness is likely not at random but more likely in the patients who are older and with more severe acute illness. In contrast, self‐reported COVID‐19 vaccination status may be easier to obtain in other populations, such as ambulatory patients or other patients in outpatient settings. For example, since the 2012–2013 influenza season, the U.S. Flu VE network has relied primarily on self‐report of influenza vaccination to estimate mid‐season VE because influenza vaccine registries have delays in reporting.^16^, ^19^, ^26^ Collecting self‐reported vaccination status may also be helpful to quickly estimate VE in outbreak settings or for large, national datasets where participants are surveyed but do not have data linked to other vaccination sources.^17^ Additionally, self‐reported vaccination is commonly used in other non‐VE studies to evaluate vaccination programs.^11^, ^27^

Both self‐report and documented vaccinations have limitations and are subject to misclassification for different reasons, but by combining the two sources of vaccination, misclassification is minimalized. A main source of misclassification is the timing of vaccination. Timing is critical for all VE analyses, but for other vaccinations like influenza, there are typically three or more months between when vaccination is recommended (by the end of October) and the seasonal influenza peak (typically January or later).^26^ In contrast, for the COVID‐19 pandemic, illnesses are occurring concurrently with vaccination especially in our analysis, where VE is assessed shortly after vaccine introduction. Thus, reliable, high‐quality data on vaccination dates are critical because a few days can change the exposure classification (e.g., from partially vaccinated to fully vaccinated). However, our COVID‐19 VE estimates were similar by self‐report (86%) and documentation (85%).

In our analysis, there were few instances (5%) where self‐report and documented COVID‐19 vaccination differed. Sixteen patients self‐reported as unvaccinated but were confirmed through source documentation to have had at least one dose. Fifty‐four patients self‐reported receiving vaccination, but no supporting source documentation was found. The most common reason for discordance among those reporting vaccination but unable to locate source documentation were those vaccinated outside of the state or country (28%, 15/54), likely reflecting out‐of‐state vaccinations not being visible in the source documents reviewed by study coordinators. The lack of a national system to link vaccination records across jurisdictions makes vaccination status difficult to determine for patients who receive vaccination in a different jurisdiction than their residence without cross‐jurisdiction coordination and, in some scenarios, additional data sharing agreements.^18^ Other differences between self‐report and source documentation included individuals vaccinated by a military healthcare program (n = 9) or through primary care or other health provider (n = 8). These vaccination locations may be delayed in vaccination submission to IIS.^18^

Among patients with discordant vaccination status, patients who were female, had a high‐school education or less, or were unemployed were more likely to have discordance between self‐report and documented COVID‐19 vaccination status. Lower education and unemployment have previously been associated with discordance between vaccination sources.^28^ There may be other factors that could explain discordance between sources such as recall bias, misclassification of vaccines, and interviewer biases. However, due to the timing of this analysis where the median time between full vaccination and study interview was 45 days, and given the global attention of COVID‐19, recall biases may be less relevant to this analysis, but analyses examining further time since vaccination and after the introduction of booster doses may be subject to these biases.^11^, ^18^ Further, interviews were conducted consistently by trained staff across sites with regular network‐wide and site‐specific meetings to maintain consistency in interviews and data entry across the network to prevent misclassification of vaccines and interview biases.

This analysis is subject to limitations. First, this analysis was limited to hospitalized patients, and results may be different in outpatients. In addition, the concordance analysis could produce different results based on the distribution of excluded patients across case and control groups. Second, the patients in the group with missing self‐report data were different than the patients included in our analysis. Among those missing self‐report data, there was a higher percentage of patients for whom documented vaccination was available, so the VE estimate for this group may be different than patients included in our self‐report VE analysis.^11^ However, VE estimates between self‐report and documented COVID‐19 vaccination were very similar further justifying the use of combined estimates to ensure selection biases are not present in these analyses. Third, sites with preexisting relationships with IIS, including sites within the IVY network, may more easily access documented vaccination data leading to a more reliable and efficient vaccine source documentation process. Fourth, in this analysis, patients were asked about vaccination status shortly after vaccination because COVID‐19 vaccines were available to all adults in the United States as of April 19, 2021.^29^ These results may have differed if patients were interviewed after more time since vaccination had passed and could differ after the introduction of booster doses and if COVID‐19 vaccines become routine. Lastly, social desirability bias may be present in our analysis, with patients reporting false vaccination status during interview. Unvaccinated patients may also report vaccination if they have concerns about stigma associated with not being vaccinated.

Among patients able to provide self‐report information on COVID‐19 vaccination status either by themselves or through a proxy, self‐report and source documented vaccination status were highly concordant in this analysis. The bias introduced by failing to capture vaccination status from the most severely ill patients must be considered in future studies, possibly through multiple imputation of missing values. Self‐report and source documentation are complementary sources of vaccine data, and combining the two sources can increase the sample size and reduce selection biases, improving VE estimates. In public health emergency settings where data on vaccine protection are needed for rapid policy decision making, self‐report alone can be used to rapidly assess real‐world and real‐time VE in scenarios where documented vaccination may take more time and resources.

## CONFLICT OF INTEREST

All authors have completed and submitted the International Committee of Medical Journal Editors form for disclosure of potential conflicts of interest. Wesley H. Self reports grant funding from CDC for this work, grants and consultant fees from Merck outside this work, and consultant fees from Aerpio Pharmaceuticals outside this work. Adit A. Ginde reports grant support from NIH, DOD, and investigator initiated grant support from AbbVie and Faron Pharmaceuticals, all outside of this work. Jonathan D. Casey reports a grant (N23HL153584) from the National Institutes of Health (NIH). D. Clark Files reports consultant fees from Cytovale and membership on a Medpace Data Safety Monitoring Board (DSMB). David N. Hager reports salary support from Incyte Corporation, EMPACT Precision Medicine, and the Marcus Foundation. Michelle N. Gong reports grant support from NIH and the Agency for Healthcare Research and Quality (AHRQ) and fees for participating on a DSMB for Regeneron and for participating on a scientific advisory panel for Philips Healthcare. Daniel J. Henning reports consulting fees from Cytovale and Opticyte. Ithan D. Peltan reports grants from NIH and Janssen Pharmaceuticals and institutional fees from Asahi Kasei Pharma and from Regeneron. Samuel M. Brown reports fees from Hamilton for chairing a DSMB and institutional fees from Faron, Sedana, and Janssen; grants from Sedana, Janssen, NIH, and the Department of Defense (DoD); book royalties from Oxford University and Brigham Young University; and personal fees from New York University for service on a DSMB. Emily T. Martin reports personal fees from Pfizer for unrelated work and a grant from Merck for unrelated work. Akram Khan reports grants from United Therapeutics, Johnson & Johnson, 4D Medical, Lung LLC, and Reata Pharmaceuticals. Steven Y. Chang was a speaker for La Jolla Pharmaceuticals and a Consultant for PureTech Health. Jennie H. Kwon reports grant support from NIH. Matthew C. Exline reports talks on nutrition in COVID pneumonia at APEN conference sponsored by Abbott Labs. Natasha Halasa reports grants from Sanofi and Quidel. James D. Chappell reports a grant from the National Center for Advancing Translational Sciences, NIH. Adam S. Lauring reports consultant fees from Sanofi and fees from Roche for membership on a trial steering committee. Carlos G. Grijalva reports consultant fees from Pfizer, Merck, and Sanofi‐Pasteur and grants from Campbell Alliance/Syneos Health, NIH, the Food and Drug Administration, AHRQ, and Sanofi. Todd W. Rice reports personal fees from Cumberland Pharmaceuticals, Inc., as the Director of Medical Affairs; consultant fees from Cytovale, Inc.; and DSMB membership fees from Sanofi. Christopher J. Lindsell reports grants from NIH, DoD, and the Marcus Foundation; organizational contract fees from bioMerieux, Endpoint LLC, and Entegrion, Inc.; and a patent issued to Cincinnati Children's Hospital Medical Center for risk stratification in sepsis and septic shock. No other potential conflicts of interest were disclosed.

## DISCLAIMER

The findings and conclusions in this report are those of the authors and do not necessarily represent the official position of the Centers for Disease Control and Prevention (CDC).

## ORISE STATEMENT

This research was supported in part by an appointment to the Research Participation Program at the Centers for Disease Control and Prevention administered by the Oak Ridge Institute for Science and Education through an interagency agreement between the U.S. Department of Energy and CDC.

## AUTHOR CONTRIBUTIONS

**Meagan Stephenson:** Conceptualization; data curation; formal analysis; investigation; methodology; project administration; validation; visualization. **Samantha Olson:** Conceptualization; data curation; formal analysis; investigation; methodology; project administration; validation; visualization. **Wesley Self:** Conceptualization; data curation; funding acquisition; investigation; methodology; project administration; resources. **Adit Ginde:** Data curation; funding acquisition; investigation; project administration; resources. **Nicholas Mohr:** Data curation; funding acquisition; investigation; project administration; resources. **Manjusha Gaglani:** Data curation; funding acquisition; investigation; project administration; resources. **Nathan Shapiro:** Data curation; funding acquisition; investigation; project administration; resources. **Kevin Gibbs:** Data curation; funding acquisition; investigation; project administration; resources. **David Hager:** Data curation; funding acquisition; investigation; project administration; resources. **Matthew Prekker:** Data curation; funding acquisition; investigation; project administration; resources. **Michelle Gong:** Data curation; funding acquisition; investigation; project administration; resources. **Jay Steingrub:** Data curation; funding acquisition; investigation; project administration; resources. **Ithan Peltan:** Data curation; funding acquisition; investigation; project administration; resources. **Emily Martin:** Data curation; funding acquisition; investigation; project administration; resources. **Raju Reddy:** Data curation; funding acquisition; investigation; project administration; resources. **Laurence Busse:** Data curation; funding acquisition; investigation; project administration; resources. **Abhijit Duggal:** Data curation; funding acquisition; investigation; project administration; resources. **Jennifer Wilson:** Data curation; funding acquisition; investigation; project administration; resources. **Nida Qadir:** Data curation; funding acquisition; investigation; project administration; resources. **Christopher Mallow:** Conceptualization; funding acquisition; investigation; project administration; resources. **Jennie Kwon:** Data curation; funding acquisition; investigation; project administration; resources. **Matthew Exline:** Data curation; funding acquisition; investigation; project administration; resources. **James Chappell:** Data curation; funding acquisition; investigation; project administration; resources. **Adam Lauring:** Data curation; funding acquisition; investigation; project administration; resources. **Adrienne Baughman:** Data curation; funding acquisition; investigation; project administration; resources. **Christopher Lindsell:** Data curation; funding acquisition; methodology; project administration; resources. **Kimberly Hart:** Data curation; funding acquisition; investigation; project administration; resources. **Nathaniel Lewis:** Investigation; methodology; project administration. **Manish Patel:** Conceptualization; data curation; formal analysis; funding acquisition; investigation; methodology; project administration; supervision; validation; visualization. **Mark Tenforde:** Conceptualization; data curation; formal analysis; investigation; methodology; project administration; supervision; validation; visualization.

## Supporting information


**Table S1.** Characteristics of patients excluded from concordance analysis due to missing self‐reported vaccination compared to patients included in analysis, 18 US medical centers, March 11–June 6, 2021

## Data Availability

The data that support the findings of this study are available from the corresponding author upon reasonable request. The data are not publicly available due to privacy or ethical restrictions.

## APPENDIX A IVY NETWORK INVESTIGATORS

Baylor Scott & White Health: Nicole Calhoun, Kempapura Murthy, Tresa McNeal, Shekhar Ghamande, Judy Herrick, Amanda McKillop, Eric Hoffman, Martha Zayed, Michael Smith, Natalie Settele, Jason Ettlinger, Elisa Priest, Jennifer Thomas, Alejandro Arroliga, Madhava Beeram. Baystate Medical Center: Ryan Kindle, Lori‐Ann Kozikowski, Lesley De Souza, Scott Ouellette, Sherell Thornton‐Thompson. Beth Israel Deaconess Medical Center: Patrick Tyler. Cleveland Clinic: Omar Mehkri, Meg Mitchell, Connery Brennan, Kiran Ashok, Bryan Poynter. Emory University: Caitlin C. ten Lohuis, Nicholas Stanley. Hennepin County Medical Center: Heidi L. Erickson, Audrey Hendrickson, Ellen Maruggi, Tyler Scharber. Intermountain Medical Center: Samuel M. Brown, Jeffrey Jorgensen, Robert Bowers, Jennifer King, Valerie Aston, Brent Armbruster. Johns Hopkins Hospital: Arber Shehu, Richard E. Rothman. Montefiore Healthcare Center: Amira Mohamed, Rahul Nair, Jen‐Ting (Tina) Chen. Ohio State University: Sarah Karow, Emily Robart, Paulo Nunes Maldonado, Maryiam Khan, Preston So, Madison So, Elizabeth Schwartz, Mena Botros. Stanford University: Alexandra June Gordon, Joe Levitt, Cynthia Perez, Anita Visweswaran, Jonasel Roque. University of California, Los Angeles: Steven Y. Chang, Adreanne Rivera, Trevor Frankel. UCHealth University of Colorado Hospital: Michael P. Tozier, David Huynh, Michelle Howell, Jennifer Friedel, David Douin, Conner Driver, Michael Carricato, Alexandra Foster. University of Iowa: Anne Zepeski, Paul Nassar, Lori Stout, Zita Sibenaller, Alicia Walter, Jasmine Mares, Logan Olson, Bradley Clinansmith. University of Miami: Carolina Rivas, Hayley Gershengorn, EJ McSpadden, Rachel Truscon, Anne Kaniclides, Lara Thomas, Ramsay Bielak, Weronika Damek Valvano, Rebecca Fong, William J. Fitzsimmons, Christopher Blair, Andrew L. Valesano, Julie Gilbert. Oregon Health & Science University: Catherine L. Hough, Akram Khan, Olivia Krol, Zachary Zouyed, Emma Silverman, Genesis Briceno, Emmanuel Mills. University of Washington: Daniel J. Henning, Christine D. Crider, Kyle A. Steinbock, Thomas C. Paulson, Layla A. Anderson. Vanderbilt University Medical Center: H. Keipp Talbot, Jonathan D. Casey, Natasha Halasa, Carlos G. Grijalva, Todd W. Rice, Jillian P. Rhoads, William B. Stubblefield, Kelsey N. Womack, Yuwei Zhu, Christy Kampe, Jakea Johnson, Rendie McHenry, Marcia Blair, Douglas Conway. Wake Forest University Baptist Medical Center: D. Clark Files. Wake Forest University: Mary LaRose, Leigha Landreth, Madeline Hicks, Lisa Parks. Washington University: Hilary M. Babcock, Jahnavi Bongu, David McDonald, Candice Cass, Sondra Seiler, David Park, Tiffany Hink, Meghan Wallace, Carey‐Ann Burnham, Olivia G. Arter. CDC COVID‐19 Response Team: Stephanie J. Schrag, Miwako Kobayashi, Jennifer R. Verani.
