# Supplementary material for: Ascertainment of vaccination status by self‐report versus source documentation: Impact on measuring COVID‐19 vaccine effectiveness
Source: Influenza Other Respir Viruses. 2022 Jul 11;16(6):1101–11. doi: 10.1111/irv.13023 (PMC9350035; doi:10.1111/irv.13023)
Supplement: Supplementary file 1 — Table S1. Characteristics of patients excluded from concordance analysis due to missing self‐reported vaccination compared to patients included in analysis, 18 US medical centers, March 11–June 6, 2021 [file IRV-16-1101-s001.docx]

**Supplement**

| **Table S1. Characteristics of patients excluded from concordance analysis due to missing self-reported vaccination compared to patients included in analysis, 18 US medical centers, March 11–June 6, 2021** | | | |
| --- | --- | --- | --- |
|  | **Patients without self-report information available for analysis** | **Patients included in concordance analysis** | **p-value**^ⴕ^ |
| **Total** | 594 | 1924 |  |
| **Race/Ethnicity; No. / Total no. (%)** |  |  | <0.01 |
| White, non-Hispanic/Latino | 303/594 (51) | 1111/1924 (58) |  |
| Black, non-Hispanic/Latino | 141/594 (24) | 430/1924 (22) |  |
| Hispanic/Latino | 100/594 (17) | 271/1924 (14) |  |
| Other, non-Hispanic/Latino | 31/594 (5) | 92/1924 (5) |  |
| Unknown | 19/594 (3) | 20/1924 (1) |  |
| **Age; Median (IQR)** | 61 (49-71) | 58 (45-67) | <0.01 |
| **Age Group; No. / Total no. (%)** |  |  | <0.01 |
| 18-49 | 149/583 (26) | 602/1918 (31) |  |
| 50-64 | 187/583 (32) | 677/1918 (35) |  |
| 65+ | 247/583 (42) | 639/1918 (33) |  |
| **Interviewee Type; No. / Total no. (%)** |  |  | <0.01 |
| Patient | 199/594 (34) | 1654/1924 (86) |  |
| Surrogate | 13/594 (2) | 121/1924 (6) |  |
| Mix of Patient and Surrogate | 6/594 (1) | 38/1924 (2) |  |
| No Interview | 359/594 (60) | 97/1924 (5) |  |
| Not specified | 17/594 (3) | 14/1924 (1) |  |
| **ICU Admission; No. / Total no. (%)** |  |  | <0.01 |
| Yes | 147/594 (25) | 322/1924 (17) |  |
| No | 257/594 (43) | 986/1924 (51) |  |
| Unknown | 190/594 (32) | 615/1924 (32) |  |
| **Length of Stay, days; Median (IQR)** | 5 (3-9) | 5 (3-9) | 0.65 |
| **Documented Vaccine Status** |  |  | 0.07 |
| Documented Vaccination | 258/593 (44) | 756/1924 (39) |  |
| Unvaccinated | 335/593 (56) | 1168/1924 (61) |  |

Abbreviations: IQR: Interquartile range

^ⴕ^Chi-square test used for statistical comparison.
